# Association of Cyclosporine Dose with Early Onset Hypertension in Allogeneic Hematopoietic Cell Transplant Patients: A Cohort Study

**DOI:** 10.3390/jcm15093491

**Published:** 2026-05-02

**Authors:** Yves Soltermann, Jérémie Héritier, Helen Baldomero, Jakob R. Passweg, Martina Kleber

**Affiliations:** 1 Divisions of Hematology, University Hospital Basel, 4031 Basel, Switzerland; 2 Faculty of Medicine, University of Basel, 4056 Basel, Switzerland; 3 Department of Internal Medicine, Clinic Hirslanden Zurich, 8032 Zurich, Switzerland

**Keywords:** allogeneic HCT, GVHD prophylaxis, cyclosporine A, hypertension

## Abstract

**Background:** Cyclosporine A (CsA) is a cornerstone in graft-versus-host disease (GVHD) prophylaxis in allogeneic hematopoietic cell transplantation (allo-HCT), and a higher starting dose of 5 vs. 3 mg/kg reduces the risk of acute GVHD. Since hypertension is a relevant side effect of CsA, data on whether a higher CsA starting dose affects the incidence of hypertension are warranted. **Methods**: In this monocentric cohort study, 367 patients with no preexisting hypertension and treated with a CsA-containing GVHD prophylaxis were included: 230 (63%) with a CsA starting dose of 3 mg/kg and 137 (37%) with 5 mg/kg. The primary outcome was the incidence of early new-onset hypertension during the engraftment period. Potential risk factors for early new-onset hypertension were assessed using uni- and multivariable Cox regression models. **Results**: Overall, the cumulative incidence of early new-onset hypertension was 67% (246/367), but the incidence rate for early new-onset hypertension in the higher CsA group was lower (CsA 5 vs. 3 mg/kg: 57 vs. 67 per 1,000 patient-days; *p* = 0.414). In the multivariable analysis, risk factors for early new-onset hypertension were advanced patient age, obesity and prior autologous HCT, while a higher CsA starting dose was not associated with increased early new-onset hypertension (adjusted hazard ratio, 0.90; 95% CI, 0.67–1.21). **Conclusions:** A higher CSA starting dose of 5 vs. 3 mg/kg did not increase the risk of hypertension. Since previous analyses demonstrated a reduction in GVHD with a higher CsA starting dose of 5 mg/kg, current findings further support the safety of a higher CsA starting dose.

## 1. Introduction

Allogeneic hematopoietic cell transplantation (allo-HCT) is a potentially curative treatment for many hematological malignancies [1,2,3,4,5,6,7] with relevant mortality and morbidity [3,8,9]. Important causes of non-relapse related mortality (NRM) are graft-versus-host disease (GVHD), followed by infections, toxicities and cardiovascular complications in long-term survivors [8,10]. For GVHD prophylaxis, calcineurin inhibitors (CNI) are a cornerstone, with cyclosporine A (CsA) being the most commonly used agent [11]. We had previously demonstrated that higher CsA levels on day + 10 after allo-HCT were associated with a significantly reduced incidence of acute GVHD Grade ≥ 2 [12]. Therefore, at our institution, CsA starting doses were increased from 3 mg/kg to 5 mg/kg to reach the targeted drug levels more rapidly and reliably, thereby reducing the incidence of acute GVHD Grade ≥ 2 [13].

In the setting of solid organ transplantation, CsA is known to increase blood pressure in a dose-dependent manner within days after initiation [14,15,16,17,18,19]—thus, valid comparative evidence in allo-HCT is warranted [20].

We therefore aimed to (i) evaluate the incidence rate of early new-onset hypertension in groups stratified by CsA starting dose, (ii) estimate the association of 5 mg/kg versus 3 mg/kg CsA starting doses with incident early new-onset hypertension, and (iii) identify risk factors for early new-onset hypertension.

## 2. Materials and Methods

### 2.1. Patient Population and Study Design

This monocentric cohort study included consecutive patients undergoing an allogeneic hematopoietic cell transplantation (allo-HCT) at the Division of Hematology of the University Hospital Basel from January 2010 to June 2020.

Patients over the age of 18 years receiving their first allo-HCT due to myeloid or lymphoid malignancies were included, with a CsA-based GVHD-prophylaxis starting prior to the transplantation day, with no preexisting hypertension in their patient history, and with peripheral blood or bone marrow as the graft source.

From June 2016, all included patients received CsA with a starting dose of 5 mg/kg. Prior to June 2016, our CsA starting dose was 3 mg/kg as reported previously [13].

Patient, disease and transplant characteristics were extracted from our database. Diagnosis of diabetes, dyslipidemia and chronic kidney disease (CKD) followed current guidelines [21,22,23]. The Ethics Committee of Northwestern and Central Switzerland approved this study (EKNZ 2020-01386) on 15 June 2020. All patients provided their informed consent for using their data for research purposes.

### 2.2. Conditioning Regimen and GVHD Prophylaxis

Myeloablative conditioning regimens (MAC) consisted of cyclophosphamide in combination with busulfan, cyclophosphamide (Cy) and total body irradiation (TBI) ≥ 8 Gy, carmustine, etoposide, cytarabine, melphalan with or without fludarabine (Flu) (BEAM ± Flu) and others (Flu/melphalan/cytarabin/TBI, Cy/etoposide/TBI, Cy/etoposide, Cy/Flu/TBI, Flu/TBI [6 Gy], TBI). Reduced intensity conditioning (RIC) regimens included fludarabine with low-dose TBI (<6 Gy), fludarabine combined with busulfan or melphalan and other protocols.

GVHD prophylaxis in patients with MAC consisted either of CsA (initial dose 3 mg/kg or 5 mg/kg) and methotrexate (MTX; 15 mg/m^2^), CsA and mycophenolate mofetil (MMF; 30 mg/kg) or others (CsA/MTX/MMF or CsA/Alemtuzumab). Antithymocyte globuline (ATG) was added in allo-HCT with unrelated donors and related donors, donor age ≥ 40 years, and omitted in patients with active leukemia at the time of transplantation.

In patients with RIC, GVHD prophylaxis included CsA and MTX, and CsA and MMF. For unrelated donors and matched related donors ≥ 40 years, ATG was used if the conditioning regimen was based on Flu/Bu and no enhanced graft versus leukemia effect was required [24,25]. For patients with Flu/low-dose TBI conditioning (as well as in one patient with reduced-dose BEAM protocol), no ATG was administered. Dosage of ATG was increased incrementally over 3 days (day-3, 5 mg/kg; day-2, 10 mg/kg; day-3, 20 mg/kg) [24].

The initial CsA dose was either 3 mg/kg or 5 mg/kg intravenous (i.v.) per day, starting on day-3 or on day-1 (in case of conditioning with Flu/low-dose TBI, BEAM, BEAM-Flu) before allo-HCT (allo-HCT = day 0). CsA administration was changed from i.v. to oral if reliable oral intake was possible. CsA in the Flu/low-dose TBI conditioning protocol was administered orally from the beginning.

CsA trough levels were measured weekly by high-performance liquid chromatography (HPLC) and two days after dose changes. The targeted CsA levels were between 150 and 200 µg/L [11].

### 2.3. Definition of Hypertension After CsA Application

Standardized blood pressure measurement by electronic or manual upper-arm devices during the nurse’s routine or at request was averaged to receive a mean daily blood pressure value. Blood pressure was analyzed from the first day of CsA application until engraftment (first of two consecutive days with an absolute neutrophil count (ANC) ≥ 0.5 × 10^9^/L). Early new-onset hypertension, our primary outcome, has been ascertained for patients without history of hypertension prior to CsA administration fulfilling the following criteria: (i) at least one mean daily blood pressure value from the day of CsA application to engraftment meeting the hypertension criteria according to the ESC guidelines 2018, (ii) and/or a new antihypertensive therapy (for at least 3 consecutive days) had to be prescribed for blood pressure control [26,27]. Primarily, hypertension is presented according to the ESC Guidelines 2018 [26]. For sensitivity analyses, we also defined hypertension according to the ACC guidelines 2017 [27].

### 2.4. Statistical Analysis

Continuous variables were expressed as mean (standard deviation [SD]) or median (interquartile range [IQR]), depending on the data distribution. We reported frequencies and percentages for categorical variables. We assessed differences in demographic, clinical, and transplantation parameters using Chi-squared or Fisher’s exact tests for categorical variables and the unpaired *t*-test or Mann–Whitney U test for numerical variables, as appropriate.

For the calculation of incidence rates per 1,000 patient-days during the study period, the numerator was the total count of early new-onset hypertension cases, and the denominator was the total patient-days at risk for early new-onset hypertension. Follow-up was calculated from the first day of CsA administration to early new-onset hypertension, death, or engraftment, whichever occurred first. Probabilities of early new-onset hypertension were compared between the two CsA starting dose groups with the Kaplan–Meier estimator. For analysis of potential risk factors for early new-onset hypertension, we fitted univariable and multivariable Cox regression models. Given the etiological study question and potential competing events, a cause-specific modeling approach was chosen. Variables considered were CsA starting dose (main exposure; 5 mg/kg, 3 mg/kg), age, sex, smoking status (never, former, current), diabetes, obesity (no versus grade 1–3), dyslipidemia, CKD, number of prior autologous transplantations, conditioning intensity, conditioning regimens with TBI and antifungal treatment. Our analyses relied on standard software (SPSS Statistics v22, IBM, Chicago, IL, USA and Stata SE v15; StataCorp LLC, College Station, TX, USA). All reported *p*-values are two-sided.

## 3. Results

### 3.1. Patient Characteristics

**Figure 1:**
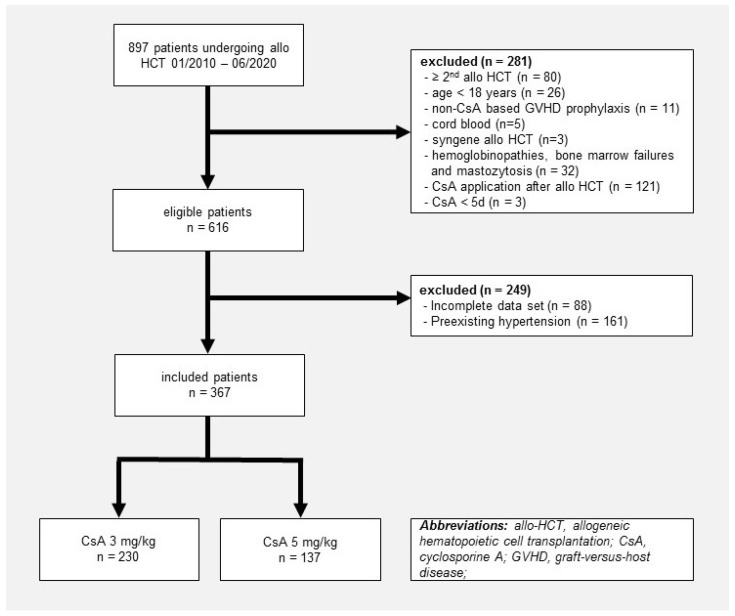
Selection chart.

During the study period, 367 patients met the study inclusion criteria: 230 (63%) patients with a CsA starting dose of 3 mg/kg and 137 (37%) patients with a CsA starting dose of 5 mg/kg for GVHD prophylaxis, as shown in the selection chart (Figure 1). Patients with preexisting hypertension were excluded from the primary analysis to allow a valid assessment of new-onset hypertension, as only patients at risk at baseline can contribute to an incidence analysis. Patient and transplant characteristics of all patients and stratified by CsA starting dose are summarized in Table 1. Median age, donor/recipient sex and graft source were comparable in both CsA starting doses. Patients in the CsA starting dose of 5 mg/kg group versus 3 mg/kg group had a significantly higher DRI, more frequently an unrelated matched donor, as well as more CMV-positive donors. Underlying diagnoses were pronounced myeloid malignancies. A TBI containing conditioning was less common in the CsA 5 mg/kg versus the CsA 3 mg/kg group. Furthermore, patients in the CsA 5 mg/kg group, in contrast to those in the CsA 3 mg/kg group, received more often reduced-intensity conditioning and had more frequently an ATG-containing GVHD prophylaxis, along with MTX also being more frequently used. Follow-up until censoring (calculated with inverse Kaplan–Meier method) was median 19 days (IQR 17–22), 21 days (IQR 18–24), and 18 days (IQR 17–21) for all patients vs. patients in the CsA 5 and 3 mg/kg group, respectively. Cardiovascular risk factors at baseline in all patients and according to CsA starting dose are depicted in Table 2. These were similar between the CsA starting dose 5 mg/kg group and the CsA starting dose 3 mg/kg group, except for sex (male 64% vs. 53%, respectively; *p* = 0.030) and dyslipidemia, respectively (18% vs. 28%; *p* = 0.025). Median CsA trough levels were significantly higher in the 5 mg/kg starting dose group compared to the 3 mg/kg starting dose group at day 2–4 (149 vs. 135 µg/L, *p* = 0.03) and at day 10 (203 vs. 181 µg/L, *p* < 0.001).

### 3.2. Early New-Onset Hypertension

The cumulative incidence of early new-onset hypertension in the study population was 67% (246/367). For the primary outcome ascertained by the ESC and ACC criteria (sensitivity analysis), there was no significant difference in the incidence rate of early new-onset hypertension in patients with CsA starting dose of 5 mg/kg vs. 3 mg/kg (Table 3).

### 3.3. Univariable and Multivariable Analysis of Hypertension Risk Factors

Univariable analysis revealed older patient age (HR, 1.02; 95% CI, 1.01–1.03; *p* < 0.001) and four prior autologous HCT (HR, 10.06; 95% CI, 1.38–73.37; *p* = 0.023) as potential risk factors for early new-onset hypertension after allo-HCT (Table 4). Of note, no association between a higher CsA starting dose of 5 mg/kg vs. 3 mg/kg and incident hypertension was evident, which was also confirmed in multivariable analysis.

Multivariable analysis verified the univariable analysis results regarding higher age (HR, 1.03; 95% CI, 1.02–1.04; *p* < 0.001) and four prior autologous HCT (HR, 8.37, 95% CI, 1.09–64.13, *p* = 0.041). Further, patients with obesity grade 1 had significantly higher hazards for early new-onset hypertension compared to non-obese patients (HR, 1.55; 95% CI, 1.03–2.34, *p* = 0.038). Sex, smoking, dyslipidemia, CKD, conditioning, use of TBI, and antifungal treatment were not associated with early new-onset hypertension in multivariable analysis (Table 4).

## 4. Discussion

GVHD remains a relevant cause for non-relapse related mortality after allo-HCT [8,9,10,28]. Optimizing GVHD prophylaxis is one approach to reduce the risk in this special patient cohort. Several published studies demonstrated an impact of higher CsA levels on the incidence of aGVHD [12,13,29,30,31]. In previous studies from our group, we showed that by increasing the CsA starting dose from 3 mg/kg to 5 mg/kg, a higher proportion of patients achieved targeted CsA levels [12] early after allo-HCT, resulting in a reduced incidence of aGVHD [13]. Despite higher CsA exposure in the 5 mg/kg starting group, no significant increase in the incidence of early new-onset hypertension was observed.

The implementation of a higher CsA starting dose may induce potential side effects during the early phase after allo-HCT. In solid organ transplantation, published studies demonstrated CsA as a dose-dependent risk factor for hypertension [14,15,16,17,19]. Induced by multiple mechanisms, including activation of the renin–angiotensin–aldosterone system (RAAS), sympathetic nervous system, endothelin system, and impaired responsiveness to vasodilators such as prostacycline and nitric oxide, CsA raises blood pressure through both vasoconstriction in the afferent arteriole of the glomeruli and systemic vasoconstriction [14,19,32]. Of note, during the early allo-HCT period, there are only limited data from small cohorts for the incidence of hypertension, its risk factors and the influence of CsA dosing. Therefore, further evidence is warranted [20,33,34].

Our study analyzed the incidence rate and its potential risk factors of early new-onset hypertension defined by ESC and ACC guidelines occurring from the first day of CsA administration until engraftment in solely adult patients undergoing allo-HCT and being treated with a CsA starting dose of 3 mg/kg or 5 mg/kg.

Overall incidence of early new-onset hypertension after CsA application in our cohort of patients without preexisting hypertension was 67% (246/367). However, a higher CsA starting dose of 5 mg/kg compared to 3 mg/kg did not affect its incidence rate, indicating that a higher CsA starting dose of 5 mg/kg may not further increase the risk of hypertension.

In a comparable study, Majhail et al. analyzed the incidence of hypertension in a combined cohort of adults and children after allo-HCT, all receiving a CsA-based GVHD prophylaxis, with a follow-up time of 2 years [20]. In this retrospective single-center study including 180 patients (104 adults and 76 children), with mainly hematopoietic neoplasms as the underlying disease (71%), hypertension was defined according to the National Heart, Lung and Blood Institute (NHLBI) guidelines for adults and children, which were in effect at the time of publication [35]. For adult patients, this was in line with the ESC 2018 guidelines [26] used in our study. Patients were classified as hypertensive with two abnormal blood pressure measurements or when treated with antihypertensive medication [20]. New-onset hypertension developed in 49% of patients within 4 weeks after allo-HCT; the proportion of adult patients was not stated. Further, CsA exposure post-transplant during the study period of 2 years was identified as the sole predictive factor for the development of new-onset hypertension in multivariable analysis [20].

In contrast to Majhail et al., our study did not demonstrate an association between different CsA starting doses (5 mg/kg vs. 3 mg/kg) and the development of hypertension during the early post-transplant period. The respective uni- and multivariable results were consistent and not substantially affected by the modeled baseline and transplant-related confounders. Our study was specifically designed to assess the impact of different CsA starting doses as an early adverse event, building on prior observations suggesting that a higher CsA starting dose may improve drug exposure [13]. In this context, we have now aimed to evaluate whether an increased CsA starting dose is associated with a higher risk of early hypertension.

The differences compared to previous studies are likely explained by the different time frames and exposure definitions. While Majhail et al. identified CsA use during the follow-up of 2 years as a potential risk factor for hypertension, our analysis focuses on the early post-transplant phase until engraftment. Of note, hypertension has been shown to occur frequently within the first weeks after allo-HCT and may partially resolve over time [20], suggesting a dynamic and potentially transient process, indicating that early hypertension may be driven by peri-transplant factors, such as acute pharmacological effects, fluid shifts, and concomitant medications, rather than initial CsA dosing. This may indicate a minor role of mean CsA dosing levels during the early phase after allo-HCT in the development of hypertension. Nevertheless, CsA exposure over the long term seems to significantly influence the incidence of hypertension [20].

In another retrospective single-center study, with 157 pediatric patients, all treated with CsA as GVHD prophylaxis by Kwon et al., 38% of patients developed hypertension within the first 28 days after allo-HCT [33]. Patients with preexisting hypertension or a predisposing condition (chronic kidney disease, diabetes mellitus) were not included in the analysis [33]. Similarly, in our cohort, patients with preexisting hypertension were excluded from the primary analysis to allow for a valid assessment of the hypertension incidence. Blood pressure values were assessed on five evenly distributed days during the first 28 days after allo-HCT. Patients were hypertensive when at least one average blood pressure met the definition criteria of hypertension according to NHLBI guidelines for children and adolescents. In the multivariable analysis, mean serum CsA levels until day 28 after allo-HCT were not associated with the development of hypertension, which seems to be in line with our findings [33]. Although the study period and focus on new-onset hypertension are consistent with our methodology, comparability with our results is limited due to the inclusion of only pediatric patients in the study cohort.

Tamaki et al. analyzed the impact of blood pressure within the first 28 days after allo-HCT on overall survival (OS) and NRM in 352 adult patients [34]. In 88% of the cohort, GVHD prophylaxis was based on a CsA-containing regimen. The mean blood pressure until day 28 post allo-HCT was calculated by averaging all daily mean blood pressure values. By receiver operating characteristic curve, an average systolic blood pressure cut-off of 131 mmHg was defined to predict OS and NRM. In total, 35% of the patient cohort was above the systolic blood pressure cut-off of 131 mmHg [34]. Since the established cut-off was approximately in line with hypertension criteria according to ACC 2017 guidelines [27], a comparison with our results was possible. Further, patients with preexisting hypertension were not excluded from the analysis, and a minority of patients received tacrolimus-containing GVHD prophylaxis [34].

The discussed studies underline the high frequency of hypertension early after allo-HCT [20,33,34] and suggest CsA exposure as a relevant risk factor for hypertension in long-term follow-up. Whether early new-onset hypertension after allo-HCT is associated with higher long-term cardiovascular risk profiles remains to be evaluated in future studies.

Within our study, we performed a multivariable analysis of risk factors for new-onset hypertension. The results revealed that older patient age, four prior autologous HCT and obesity grade 1 significantly increased the risk of early new-onset hypertension, while a higher CsA starting dose of 5 mg/kg did not increase the risk of early new-onset hypertension. Older patient age and obesity are well-established risk factors for hypertension [36]. A high number of prior therapies such as autologous HCT may reflect toxic effects on the vasculature, especially endothelial cells, making patients more susceptible to developing hypertension [34,37,38,39]. Obesity grades 2 and 3 did not reach statistical significance, probably due to small case numbers. Steroid exposure was primarily associated with ATG-containing regimens and was therefore captured by including ATG use in the respective uni- and multivariable models. The results did not identify an effect of ATG use or antifungal therapy on the development of early new-onset hypertension.

The strength of our study is that we analyzed new-onset hypertension according to ESC and ACC guidelines in adult patients undergoing allo-HCT receiving CsA at different dose intensities. Considering the retrospective nature of our study and comparing two consecutive study periods (CsA 3 mg/kg 2010–2016; CsA 5 mg/kg 2016–2020), differences in patient and transplant characteristics were unavoidable. These differences also reflect trends in allo-HCT over the past decade (more unrelated donors, higher DRI, more RIC, fewer lymphoid malignancy receiving allo-HCT) [2,40,41,42]. We cannot exclude confounding by unmeasured variables, as the different starting doses of CsA occurred in different years. We tried to carefully adjust for all measured variables including ATG use in multivariable analysis. Additionally, due to the retrospective study design, further detailed and standardized data on concomitant medications were not consistently available beyond selected potential risk factors such as ATG use and antifungal therapy. Thus, potential effects of other co-medications and drug–drug interactions on blood pressure and consequent unmeasured confounding in our primary analysis cannot be excluded.

Given our extended study period and retrospective design with a fixed number of available patients, our primary analysis has not been informed by a formal sample size or power calculation. Given the respective 95% confidence intervals, we cannot exclude small effects of CsA dose on hypertension incidence in the early post-transplantation phase. This would require a large, multicentric observational or interventional study.

In conclusion, we observed high cumulative incidences and incidence rates of new-onset hypertension in patients with CsA GVHD prophylaxis early after allo-HCT, especially with advanced patient age, obesity and prior intensive therapy such as auto-HCT. However, a higher CsA starting dose of 5 mg/kg compared to 3 mg/kg was not associated with early new-onset hypertension [13], underlining the strategy of using a higher CsA starting dose of 5 mg/kg as GVHD prophylaxis compared to the currently recommended 3 mg/kg [11].

## Figures and Tables

**Table 1:** Patient- and transplant-related characteristics of all patients (n = 367) and within CsA starting dose 5 mg/kg (n = 137) versus CsA starting dose 3 mg/kg (n = 230).

Variable	All Patients (n = 367)	CsA 5 mg/kg Group (n = 137)	CsA 3 mg/kg Group (n = 230)	*p*-Values
	n (%)	n (%)	n (%)	
**Age (years, median (IQR))**	51 (41–61)	50 (39–61)	51 (42–61)	0.677
**Diagnosis**				<0.001
Myeloid malignancy	251 (68)	111 (81)	140 (61)	
Lymphoid malignancy	116 (32)	26 (19)	90 (39)	
**Disease Risk Index**				0.003
Low	68 (19)	22 (16)	46 (20)	
Intermediate	183 (50)	59 (43)	124 (54)	
High	103 (28)	46 (34)	57 (25)	
Very High	13 (4)	10 (7)	3 (1)	
**Conditioning regimen**				0.002
Myeloablative	240 (65)	76 (55)	164 (71)	
Reduced intensity	127 (35)	61 (45)	66 (29)	
**TBI**				0.008
Yes	99 (27)	26 (19)	73 (32)	
No	268 (73)	111 (81)	157 (68)	
**GvHD prophylaxis**				<0.001
CsA/MTX	300 (82)	129 (94)	171 (74)	
CsA/MMF	63 (17)	4 (3)	59 (26)	
Others	4 (1)	4 (3)	0 (0)	
ATG used	215 (59)	107 (78)	108 (47)	<0.001
**Donor/recipient sex**				0.276
Female/female	71 (19)	21 (15)	50 (22)	
Female/male	83 (23)	28 (20)	55 (24)	
Male/female	72 (20)	31 (23)	41 (18)	
Male/male	141 (38)	57 (42)	84 (37)	
**Donor/recipient CMV status**				0.006
Negative/negative	113 (31)	39 (28)	74 (32)	
Negative/positive	80 (22)	19 (14)	61 (27)	
Positive/negative	39 (11)	20 (15)	19 (8)	
Positive/positive	135 (37)	59 (43)	76 (33)	
**Donor type**				<0.001
Related matched	146 (40)	54 (39)	92 (40)	
Unrelated matched	179 (49)	80 (58)	99 (43)	
Unrelated mismatched	42 (11)	3 (2)	39 (17)	
**Graft source**				0.308
Bone marrow	19 (5)	5 (4)	14 (6)	
Peripheral blood	348 (95)	132 (96)	216 (94)	

Abbreviations: ATG, antithymocyte globuline; CMV, cytomegalovirus; CsA, cyclosporine A; GvHD, graft versus host disease; IQR, interquartile range; MMF, mycophenolate mofetil; MTX, methotrexate; TBI, total body irradiation.

**Table 2:** Cardiovascular risk factors of all patients (n = 367) and within CsA starting dose 5 mg/kg (n = 137) versus CsA starting dose 3 mg/kg (n = 230) at baseline.

Variable	All Patients (n = 367)	CsA 5 mg/kg Group (n = 137)	CsA 3 mg/kg Group (n = 230)	*p*-Values
	n (%)	n (%)	n (%)	
**Sex**				0.030
male	209 (57)	88 (64)	121 (53)	
female	158 (43)	49 (36)	109 (47)	
**Smoking**				0.820
yes	176 (48)	64 (47)	112 (49)	
no	180 (49)	68 (50)	112 (49)	
unknown	11 (3)	5 (4)	6 (3)	
**Obesity**				0.793
yes	45 (12)	16 (12)	29 (13)	
no	322 (88)	121 (88)	201 (87)	
**Dyslipidemia**				0.025
Yes	88 (24)	24 (18)	64 (28)	
No	279 (76)	113 (82)	166 (72)	
**Type 2 diabetes**				0.719
yes	18 (5)	6 (4)	12 (5)	
no	349 (95)	131 (96)	218 (95)	
**Chronic kidney disease**				0.741
yes	29 (8)	10 (7)	19 (8)	
no	338 (92)	127 (93)	211 (92)	

Abbreviations: CsA, cyclosporine A.

**Table 3:** Incidence rate of early new-onset hypertension stratified by different CsA starting doses.

Variable	CsA 5 mg/kg (n = 137)	CsA 3 mg/kg (n = 230)	*p*-Values
**Incidence rate per 1,000 patient days (IQR)**			
According ESC	57 (46–70)	67 (57–78)	0.414
According ACC	120 (100–143)	145 (126–167)	0.277

Abbreviations: ACC, American College of Cardiology; CsA, cyclosporine A; ESC, European Society of Cardiology; IQR, interquartile range; Hypertension according to ESC Guidelines 2018 [26]; ACC Guidelines 2017 [27].

**Table 4:** Univariable and multivariable analysis of hypertension risk factors for early new-onset hypertension after CsA initiation, according to ESC 2018 Guidelines [26].

Variable	Univariable Analysis			Multivariable Analysis		
	HR	95% CI	*p*-Values	HR	95% CI	*p*-Values
**Age in years**	1.02	1.01–1.03	<0.001	1.03	1.02–1.04	<0.001
**Sex**						
male	Ref.			Ref.		
female	0.97	0.75–1.25	0.812	0.88	0.67–1.16	0.369
**Smoking**						
never	Ref.			Ref.		
current	0.96	0.68–1.43	0.936	0.96	0.65–1.42	0.830
former	1.02	0.79–1.39	0.754	0.91	0.67–1.23	0.523
**Obesity**						
no	Ref.			Ref.		
Grade 1	1.43	0.96–2.11	0.078	1.55	1.03–2.34	0.038
Grade 2	1.59	0.79–3.23	0.196	1.80	0.85–3.82	0.126
Grade 3	2.10	0.29–14.97	0.462	3.77	0.51–27.97	0.194
**Dyslipidemia**						
no	Ref.			Ref.		
yes	1.14	0.85–1.51	0.388	1.09	0.80–1.49	0.586
**Type 2 diabetes**						
no	Ref			Ref.		
yes	1.04	0.58–1.86	0.895	0.90	0.49–1.64	0.729
**Chronic kidney disease**						
no	Ref.			Ref.		
yes	1.15	0.73–1.82	0.549	0.85	0.52–1.42	0.540
**CsA dose**						
3 mg/kg	Ref.			Ref.		
5 mg/kg	0.90	0.70–1.17	0.431	0.90	0.67–1.21	0.477
**Conditioning intensity**						
Reduced intensity	Ref.			Ref.		
Myeloablative	0.81	0.63–1.05	0.112	1.25	0.89–1.75	0.201
**Conditioning TBI used**						
no	Ref.			Ref.		
yes	0.94	0.70–1.25	0.655	1.16	0.84–1.61	0.368
**ATG used**						
no	Ref.			Ref.		
yes	1.18	0.91–1.54	0.199	1.22	0.90–1.64	0.194
**Antifungal treatment**						
no	Ref.			Ref.		
yes	1.10	0.80–1.49	0.561	1.20	0.87–1.67	0.273
**Prior autologous HCT**						
0	Ref.			Ref.		
1	0.63	0.30–1.34	0.232	0.68	0.31–1.47	0.322
2	0.70	0.17–2.83	0.621	0.82	0.20–3.40	0.785
3	2.56	0.63–10.34	0.187	2.24	0.52–9.67	0.282
4	10.06	1.38–73.37	0.023	8.37	1.09–64.13	0.041

Abbreviations: ATG, antithymocyte globuline; CI, confidence interval; CsA, cyclosporine A; ESC, European Society of Cardiology; HCT, hematopoietic cell transplantation; TBI, total body irradiation. Obesity Grade 1, BMI 30 ≤ 35 kg/m^2^, Grade 2, BMI 35 ≤ 40 kg/m^2^, Grade 3, BMI > 40 kg/m^2^.

## Data Availability

Study data (incl. data dictionaries) are available upon request from the corresponding author if the responsible ethics committee permits the respective secondary analysis project and if high data protection standards and anonymization are guaranteed. The data are not publicly available due to privacy or ethical restrictions.

## Abbreviations

The following abbreviations are used in this manuscript:

ACC	American College of Cardiology
Allo-HCT	allogeneic hematopoietic cell transplantation
ANC	absolute neutrophil count
ATG	antithymocyte globuline
BEAM	carmustine, etoposide, cytarabine, melphalan
CI	confidence interval
CKD	chronic kidney disease
CMV	cytomegalovirus
CNI	calcineurin inhibitor
CsA	cyclosporine A
Cy	cyclophosphamide
DRI	disease risk index
ESC	European Society of Cardiology
Flu	fludarabine
GvHD	graft versus host disease
HCT	hematopoietic cell transplantation
HPLC	high-performance liquid chromatography
HR	hazard ratio
IQR	interquartile range
MAC	myeloablative conditioning
MMF	mycophenolate mofetil
MTX	methotrexate
NRM	non-relapse related mortality
OS	overall survival
RAAS	renin–angiotensin–aldosterone system
RIC	reduced intensity conditioning
SD	standard deviation
TBI	total body irradiation
